# Membrane protein extraction and purification using partially-esterified SMA polymers

**DOI:** 10.1016/j.bbamem.2021.183758

**Published:** 2021-12-01

**Authors:** Olivia P. Hawkins, Christine Parisa T. Jahromi, Aiman A. Gulamhussein, Stephanie Nestorow, Taranpreet Bahra, Christian Shelton, Quincy K. Owusu-Mensah, Naadiya Mohiddin, Hannah O'Rourke, Mariam Ajmal, Kara Byrnes, Madiha Khan, Nila N. Nahar, Arcella Lim, Cassandra Harris, Hannah Healy, Syeda W. Hasan, Asma Ahmed, Lora Evans, Afroditi Vaitsopoulou, Aneel Akram, Chris Williams, Johanna Binding, Rumandeep K. Thandi, Aswathy Joby, Ashley Guest, Mohammad Z. Tariq, Farah Rasool, Luke Cavanagh, Simran Kang, Biser Asparuhov, Aleksandr Jestin, Timothy R. Dafforn, John Simms, Roslyn M. Bill, Alan D. Goddard, Alice J. Rothnie

**Affiliations:** a College of Health & Life Sciences, Aston University, Aston Triangle, Birmingham B4 7ET, UK; b School of Biosciences, University of Birmingham, Edgbaston, Birmingham B15 2TT, UK

**Keywords:** SMALP, Solubilisation, Nanoparticle

## Abstract

Styrene maleic acid (SMA) polymers have proven to be very successful for the extraction of membrane proteins, forming SMA lipid particles (SMALPs), which maintain a lipid bilayer around the membrane protein. SMALP-encapsulated membrane proteins can be used for functional and structural studies. The SMALP approach allows retention of important protein-annular lipid interactions, exerts lateral pressure, and offers greater stability than traditional detergent solubilisation. However, SMA polymer does have some limitations, including a sensitivity to divalent cations and low pH, an absorbance spectrum that overlaps with many proteins, and possible restrictions on protein conformational change. Various modified polymers have been developed to try to overcome these challenges, but no clear solution has been found. A series of partially-esterified variants of SMA (SMA 2625, SMA 1440 and SMA 17352) has previously been shown to be highly effective for solubilisation of plant and cyanobacterial thylakoid membranes. It was hypothesised that the partial esterification of maleic acid groups would increase tolerance to divalent cations. Therefore, these partially-esterified polymers were tested for the solubilisation of lipids and membrane proteins, and their tolerance to magnesium ions. It was found that all partially esterified polymers were capable of solubilising and purifying a range of membrane proteins, but the yield of protein was lower with SMA 1440, and the degree of purity was lower for both SMA 1440 and SMA 17352. SMA 2625 performed comparably to SMA 2000. SMA 1440 also showed an increased sensitivity to divalent cations. Thus, it appears the interactions between SMA and divalent cations are more complex than proposed and require further investigation.

## Highlights

• Partially-esterified SMA polymers are effective for solubilisation of membrane proteins from different expression systems.
• Proteins in partially-esterified SMALPs can be purified by Ni^2+^ affinity chromatography, but yield is lower with SMA 1440.
• Partial esterification of SMA does not overcome the sensitivity to divalent cations.
• SMA1440 is more sensitive to magnesium ions than SMA 2000.

## Graphical abstract

**Figure:**
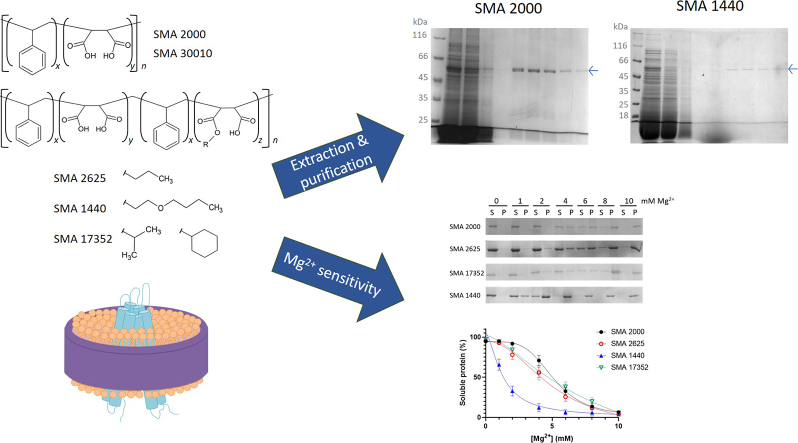

## 1 Introduction

Membrane proteins are well known to be important targets for furthering our understanding of fundamental biology and the development of novel therapeutics [1]. However, studying membrane proteins can be technically challenging due to their lipid bilayer environment. Traditional approaches have involved solubilisation of membrane proteins using mild detergents [2]. Detergents have proven successful for many proteins and downstream applications [3], but they have several limitations, including stripping away annular lipids that can be important for function, inducing protein instability due to loss of lateral pressure, and the requirement to supplement all buffers with detergent to maintain the concentration above the critical micelle concentration (CMC) [2], [4], [5], [6]. In 2009, a detergent-free alternative approach was first reported which utilises a co-polymer of styrene and maleic acid (SMA) [7]. SMA inserts into membranes and forms small discs of lipid bilayer (approx. 10 nm in diameter), with the polymer wrapped around the outside, termed SMALPs (SMA lipid particles) [8]. SMA can therefore extract membrane proteins directly from the membrane, to form a small soluble particle, with the protein retaining its lipid bilayer environment. SMA has been shown to work effectively for a wide range of different membrane proteins from many different expression systems [9], [10], [11], [12], [13], [14], [15]. SMALPs, once formed, are stable and excess free SMA can be removed. SMALP-encapsulated proteins can be purified by affinity chromatography and utilised in a wide range of downstream techniques [9], [10], [11], [12], [16], [17], [18], [19], [20], [21]. Many proteins within SMALPs have been shown to have much greater stability than when detergent solubilised [10], [11], [12], [13], [14], [22], [23], [24]. Recently, SMALP-encapsulation has been successful for structural determination of several proteins by cryo-electron microscopy [25], [26], [27], [28], [29], [30], as well as for identifying novel function or developing new assays [31], [32], [33], [34].

However, despite the many advantages offered by SMA, there are some limitations. The styrene moiety absorbs light with a λ_max_ of 260 nm [35]. This can be useful at times for monitoring and quantifying the polymer (Supplementary Fig. 1), but can be problematic for some spectroscopic approaches. SMA polymer is sensitive to pH, and precipitates out of solution at low pH [36]. This can be problematic for the study of proteins for which activity is optimal at low pH, or when they require proton binding for function. Similarly, SMA is sensitive to divalent cations such as Mg^2+^ and Ca^2+^, which also cause it, and the encapsulated protein, to precipitate out of solution [22], [23]. This is problematic for proteins which require these divalent cations for function, such as ABC (ATP Binding Cassette) transporters, which need Mg^2+^ binding for ATP hydrolysis. Finally, it has been proposed that within a SMALP some proteins may be held too tightly for full function, perhaps limiting conformational changes between states [16]. Several polymer variants have been reported that overcome some of these limitations. For example styrene maleimide (SMI) is effective at low pH and is tolerant of divalent cations [37]. SMI has been shown to extract a GPCR (G protein-coupled receptor) effectively, and has also been used to purify the membrane tether protein ZipA. However, we have to date been unable to purify an ABC transporter using SMI. SMA-QA (SMA quaternary ammonium) has been reported to be tolerant to low pH and divalent cations [38], but this polymer is not currently commercially available. The polymer DIBMA (diisobutylene maleic acid) replaces the styrene moiety with the aliphatic diisobutylene group, thus it does not absorb significantly at 260 nm [35]. DIBMA is also tolerant to divalent cations, and produces larger discs than SMA, with less restricted lipid packing [35]. The less restricted environment within DIBMA particles has been shown to allow full function of proteins which were restricted from completing all conformational changes within SMALPs [39]. However, although DIBMA works well for some proteins such as GPCRs, for others, such as the ABC transporter BmrA, it gives a lower yield of protein, lower purity and decreased stability [40], [41].

**Fig. 1:**
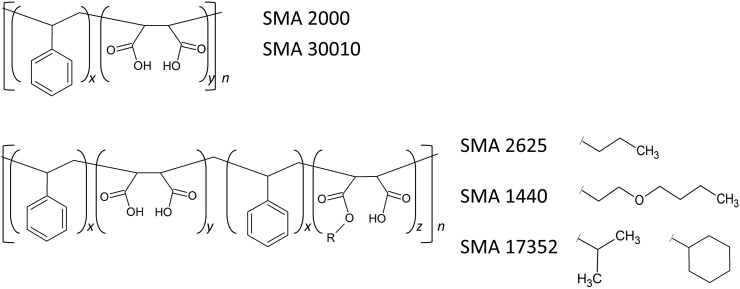
Structures of the hydrolysed polymers. SMA 2000 and SMA 30010 are co-polymers of styrene and maleic acid. SMA 2625, SMA 1440 and SMA 17352 are partially esterified variants of SMA, with the ester moieties (R) shown.

A series of partially-esterified variants of SMA has previously been tested for the solubilisation of plant and cyanobacterial thylakoid membranes [33], [42], [43], [44]. These polymers, SMA 2625, SMA 1440 and SMA 17352, have various chemical groups attached to some of the maleic acid groups of SMA (Fig. 1 & Table 1). For thylakoid membranes, these partially-esterified polymers, particularly SMA 1440, were more effective than standard SMA. However, thylakoid membranes have quite distinct properties [43] (densely packed and containing high levels of galactolipids), and the performance of these polymers has not yet been investigated for more typical phospholipid membranes from other sources. The esterification within these polymers also means they lack some of the maleic acid groups found in other polymers. These groups have been implicated in the divalent cation sensitivity of SMA, the carboxylates of the maleic acid groups being proposed to chelate Mg^2+^, inducing strain or a conformational change in the polymer surrounding the SMALP. It is thought this process causes the SMA to precipitate [16], [22]. Therefore, in this study we investigated the use of partially-esterified polymers for the solubilisation of lipids and membrane proteins, and their tolerance to magnesium ions.

**Table 1:** Properties of the polymers according to the manufacturers.

Polymer	Manufacturer	S:MA ratio	Modifications	Mw (kDa)	Mn (kDa)	PDI
SMA 2000	Cray Valley	2:1	–	7.5	3.0	2.5
SMA 30010	Polyscope	2.3:1	–	6.5	2.5	2.6
SMA 2625	Cray Valley	2:1	1-Propanol	9.0	3.6	2.5
SMA 1440	Cray Valley	1.5:1	2-Butoxyethanol	7.0	2.8	2.5
SMA 17352	Cray Valley	1.7:1	Cyclohexanol & 2-propanol	7.0	2.8	2.5

The average ratio of styrene to maleic acid within the polymers. Mw, the average molecular weight of the polymers. The number average molecular weight (Mn), which is the total weight of the polymer molecules divided by the number of molecules. The polydispersity index (PDI), which is equal to Mw/Mn and is a measure of the distribution of the molecular weights.

## 2 Methods

### 2.1 Polymer preparation

The polymers SMA 2000, SMA 1440, SMA 17352 and SMA 2625 were from Cray Valley (Exton, Pennsylvania, USA). SMA 30010 (previously called SZ30010) was from Polyscope (Geleen, Netherlands). All were supplied as the styrene maleic anhydride form, and to be used for membrane solubilisation they needed to be hydrolysed to the styrene maleic acid form. SMA 2000 and SMA 30010 were hydrolysed by reflux in 1 M NaOH as described previously [17], [22]. The partially esterified polymer variants, SMA 1440, SMA 17352 and SMA 2625 were hydrolysed using a different protocol, according to the manufacturer's recommendations. Briefly, a 10% (w/v) solution of each esterified polymer in 1 M NaOH was vigorously stirred at 50 °C for several hours, until the polymer crystals had completely dissolved. The solution was then placed in a 35 mm diameter SnakeSkin™ dialysis tubing with a molecular weight cut-off of 3.5 kDa (Thermo Fisher), and dialysed against 2 l distilled water overnight at 4 °C. The water was refreshed several times until the polymer reached pH 8. The contents of the dialysis tubing were collected and freeze dried.

### 2.2 Polymer characterisation

NMR analyses of polymers were performed on a Bruker Avance-300 spectrometer at room temperature overnight and recorded using a 5 mm normal dual detection probe. All polymer samples in the anhydride form were analysed via ^1^H NMR and ^13^C NMR. 10-50 mg of each polymer were weighed into an NMR sample tube and dissolved with approximately 0.6–1 ml of deuterated Acetone (CD_3_COCD_3_). The ^1^H NMR spectra were recorded at 300.13 MHz using a high-resolution dual (^1^H ^13^C) gradients probe. Spectra were recorded using the zg30 pulse program with 16 or 128 scans and referenced to the DMSO peak at 2.50 ppm. ^13^C NMR spectra were obtained at 75 MHz for carbon. The pendant pulse program was used with waltz16 decoupling during acquisition with 6k scans and phased for CH_3_/CH positive and quaternary carbons and CH_2_ negative with spectra referenced to DMSO peak at 30.0 ppm.

All polymers, both anhydride and acid forms, were analysed using a bench attenuated total reflectance (ATR) FTIR machine. The ATR sampling accessory crystal was cleaned with methanol and dried completely. A background scan was conducted in order to determine how much of the original IR intensity in percent transmittance was left after passing through the sample. The polymer sample in either liquid or solid forms were placed in a small drop or quantity onto the crystal and a force was applied using the ATR force arm to ensure good contact between the sample and the crystal surface. The samples were then scanned to acquire data with a scan range of 4000–650 cm^−1^.

### 2.3 Preparation of liposomes and solubilisation with polymers

DMPC (1,2-dimyristoyl-sn-glycero-3-phosphocholine) lipids (Avanti Polar Lipids) were dissolved in 2:1 chloroform: methanol and dried down under nitrogen. The lipid film was resuspended in buffer 1 (20 mM Tris pH 8, 150 mM NaCl) to form a 2% (w/v) suspension of DMPC liposomes, this was stored at 4 °C.

The rate at which DMPC liposomes were solubilised by the various SMA polymers was monitored in a 96-well plate where 100 μl of 2% (w/v) lipid was added followed by 100 μl of 2.5% (w/v) polymer in buffer 1. Light scattering was measured over time in a multiskan GO plate reader (Thermo Fisher) at a wavelength of 400 nm.

To form lipid-only SMALPs, the 2% (w/v) DMPC suspension was mixed at a 1:1 ratio with 2.5% (w/v) polymer in buffer 1, which promptly clarified (<5 s). To remove excess free polymer the sample was then run on a Superdex 200 10/300 column (GE Healthcare), equilibrated with buffer 1, at a flow rate of 0.5 ml/min, and the fractions corresponding to SMALPs harvested (Supplementary Fig. 1).

### 2.4 Dynamic light scattering (DLS)

100 μl of lipid-only SMALPs or 2% (w/v) DMPC lipid suspension was added to 1900 μl of buffer 1. Dynamic light scattering (DLS) data were recorded using a Brookhaven NanoBrook 90plus Zeta instrument (640 nm) using a 1.0 cm path length disposable cuvette (Brand BMBH). Measurements were taken at a temperature of 25 °C with 30 s equilibration time. Automated instrument parameters were used. Each measurement was repeated at least 6 times.

DLS experiments for all stability studies were performed using a DynaPro Plate Reader III and DYNAMICS software (Wyatt Technology, Haverhill, UK), using a laser wavelength of 825.4 nm with a detector angle of 150°. Each sample (40 μl of purified SMALPs or 0.625 mg/ml DMPC liposomes) was loaded into a 384-well glass bottom SensoPlate™ (Greiner Bio-One, Germany) in triplicate. Each measurement consisted of 5 scans of 5 s. The attenuator position and laser power were automatically optimised for size (nm) determination. For thermostability measurements, scans were carried out at a starting point of 25 °C, with discrete 5 °C temperature increments, up to 65 °C. Each increase in temperature was maintained to establish equilibrium before data collection. For time course studies, 100 measurements consisting of 5 acquisitions of 5 s were carried out over the course of 1 h to monitor lipid-only SMALP stability. Measurements were taken at a temperature of 25 °C.

### 2.5 Membrane protein expression & membrane preparation

ZipA, BmrA and LeuT were expressed in *E. coli* as described previously [22], [23]. Top10 *E. coli* cells were transformed with a pBAD24-GltpH_his_ vector. Small overnight cultures (5 ml) were used to inoculate 1 l flasks of Luria broth supplemented with 100 mg/ml ampicillin and grown at 37 °C, 200 rpm until OD_600_ reached 0.6. Protein synthesis was induced by the addition of 1% (w/v) arabinose, and cells were harvested 3 h later by centrifugation (6000*g*, 10 min). *E. coli* cell pellets were resuspended in buffer 2 (50 mM Tris pH 7.4, 250 mM sucrose and 0.25 mM CaCl_2_, 1 μM pepstatin, 1.3 μM benzamidine and 1.8 μM leupeptin) disrupted using either sonication (5 × 20 s bursts, on ice) or a French press (3 × 16,000 psi). A low speed spin (650*g*, 20 min) was used to remove unbroken cells and debris, then membranes were harvested by ultracentrifugation (100,000*g*, 20 min, 4 °C). MRP4/ABCC4 (multidrug resistance protein 4/ATP Binding Cassette transporter C subfamily member 4) was expressed in Sf9 insect cells, disrupted using nitrogen cavitation, and membranes harvested as described previously [45]. CD81 was expressed in *Pichia pastoris* yeast cells and membranes harvested as described previously [15]. For all proteins, membranes were resuspended in buffer 1 at 60 mg/ml (wet pellet weight) and stored in aliquots at −80 °C.

### 2.6 Solubilisation of membrane proteins

Cell membranes (60 mg/ml wet pellet weight) were mixed with an equal volume of 5% (w/v) SMA polymers, and incubated at room temperature with gentle shaking for 1 h. Samples were subjected to ultracentrifugation (100,000*g*, 20 min, 4 °C), and the soluble protein in the supernatant harvested. The insoluble material in the pellet was resuspended in an equal volume of buffer 1 supplemented with 2% (w/v) SDS. Samples of both soluble and insoluble protein were analysed by Western blotting. For most proteins an anti-polyhistidine primary antibody (R&D Systems) was used, with either an anti-mouse-HRP (Cell Signalling) or anti-mouse-alkaline phosphatase (Sigma) secondary antibody, and visualised using chemiluminescence (Pierce) and a C-Digit scanner (Licor) or colourimetrically using BCIP/NBT (Sigma). For MRP4, an anti-MRP4 M_4_I-10 primary antibody (Abcam) was used with an anti-rat-HRP secondary antibody (Sigma). The solubilisation efficiency was quantified using densitometry (Image J).

### 2.7 Ni-NTA affinity purification

Solubilised membrane proteins were mixed with washed HisPur Ni-NTA resin (ThermoFisher) at a ratio of 100 μl resin bed volume (bv) per ml of solubilised protein. This was rotated overnight at 4 °C. The mixture was poured into an empty gravity flow column (Machery-Nagel) and the flow-through collected. The resin was washed five times with 10 bv buffer 1 supplemented with 20 mM imidazole, and twice with 10 bv buffer 1 supplemented with 40 mM imidazole. Protein was eluted in 6 fractions of ½ bv each with buffer 1 supplemented with 200 mM imidazole. Samples of the wash and elution fractions were run on SDS-PAGE and stained with InstantBlue (Abcam).

Elution fractions containing purified protein were pooled, and the concentration measured by densitometric analysis of samples (5–20 μl) run on SDS-PAGE alongside BSA (bovine serum albumin) standards (0.25–1.5 μg) and stained with InstantBlue as described previously [22]. The degree of purity obtained was also assessed by densitometric analysis of lanes of SDS-PAGE gels [22].

### 2.8 Magnesium ion sensitivity assay

Purified proteins were mixed with varying concentration of MgCl_2_ (0–10 mM), then ultracentrifuged (100,000*g*, 20 min, 4 °C). The supernatant containing soluble protein was harvested, and the pellet resuspended in an equal volume of buffer. Samples of both supernatant and pellet were run on SDS-PAGE, stained with InstantBlue and analysed by densitometry to calculate the percentage remaining in solution.

Stability of lipid-only SMALPs in the presence of 0–20 mM MgCl_2_ was measured by DLS using the DynaPro Plate Reader III and DYNAMICS software, as described above.

### 2.9 Data analysis

Statistical analysis was undertaken using GraphPad Prism. An ANOVA with a Dunnett's *post-hoc* test was used for multiple comparisons; p < 0.05 was considered significant. Divalent cation sensitivity data were fitted with a normalised dose-response curve with variable slope and analysed by a two-way ANOVA with a Tukey *post-hoc* test.

## 3 Results

### 3.1 Characterisation of polymers and solubilisation of lipids

NMR characterisation of the anhydride form of each of the different polymers was undertaken, in order to determine the structure of the polymer as well as to identify the varying ester groups attached to the SMA polymer. The ^1^H NMR and ^13^C NMR spectra show peaks corresponding to various chemical environments that match with the structures shown (Supplementary Fig. 2).

To be utilised for membrane solubilisation, the maleic anhydride form of the polymer must be hydrolysed to the maleic acid form. For the partially esterified polymers this was carried out by mixing with NaOH and heating, but to lower temperatures than typically used for SMA 2000, to prevent hydrolysis of the ester groups. FTIR spectra of both the pre-hydrolysis and post-hydrolysis forms of the polymers were determined (Supplementary Fig. 3). The distinctive strong C = O bands observed at 1850 cm^−1^ and 1770 cm^−1^ in all the SMA anhydride spectra (Supplementary Fig. 3A) largely disappear in the hydrolysed SMA acid spectra (Supplementary Fig. 3B), where strong bands between 1400 and 1550 cm^−1^, indicating the stretching vibrations of carboxylate ions (COO^−^), are instead observed. However, it is notable that the hydrolysed partially-esterified polymers, retain a band at 1700 cm^−1^ that is not present in the hydrolysed SMA 2000 spectra, corresponding to the C = O ester bonds.

**Fig. 2:**
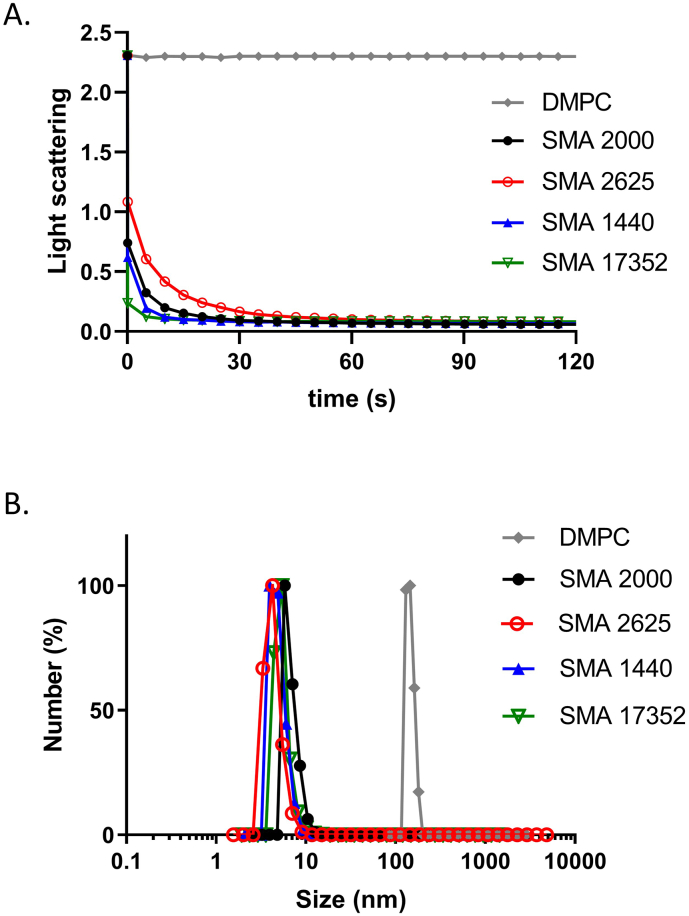
Partially esterified polymers solubilise lipids quickly and form small polymer-lipid particles. A; DMPC liposomes 2% (w/v) were mixed with an equal volume of 2.5% (w/v) polymer in buffer 1, total volume 200 μl. Light scattering was measured over time (up to 5 min), in a multiskan GO plate reader at a wavelength of 400 nm. B; Particles formed were analysed by a Brookhaven NanoBrook 90plus Zeta instrument (640 nm) with 1.0 cm path length disposable cuvettes. Lipid-only SMALPs or DMPC liposomes (100 μl) was added to 1900 μl of buffer 1. Measurements were taken at a temperature of 25 °C with 30 s equilibration time. Automated instrument parameters were used. Each measurement was repeated at least 6 times.

The ability of each polymer to solubilise lipids from liposomes was investigated next. As shown in Fig. 2A, all of the polymers solubilised the lipids very quickly, with the light scattering signal provided by the liposomes being lost completely within 30 s of polymer addition. The size of the particles formed was analysed by DLS (Fig. 2B). All of the polymers formed particles of just under 10 nm in diameter. The stability of the SMALPs was then tested further using DLS. The size of the particles remained steady over 50 min (Supplementary Fig. 4A). They were also stable at increasing temperatures up to 55 °C (Supplementary Fig. 4B), but at temperatures above this they seemed to aggregate.

### 3.2 Solubilisation of membrane proteins

**Fig. 3:**
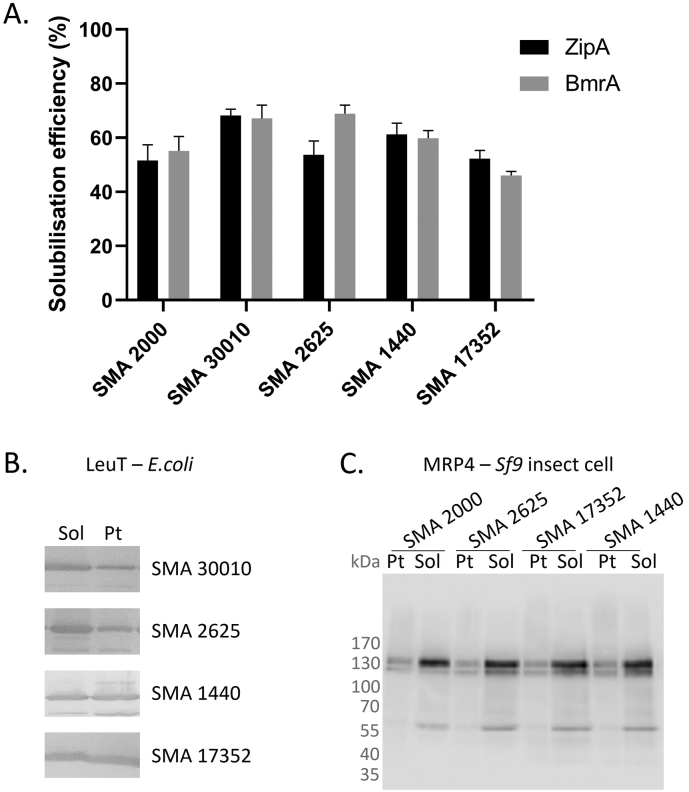
Partially-esterified polymers are effective for solubilising a range of different membrane protein families from different expression systems. Membranes (60 mg/ml wet weight) from *E. coli* expressing ZipA (A), BmrA (A) or LeuT (B), or from Sf9 insect cells expressing MRP4 (C) were mixed with 2.5% (w/v) SMA polymers for 1 h at room temperature. Following ultracentrifugation (100,000*g*, 20 min, 4 °C) samples of the soluble protein in the supernatant (Sol) and the insoluble protein in the pellet (Pt) were analysed by Western blotting using an anti-his antibody (A & B) or an anti-MRP4 M_4_I-10 antibody (C). The solubilisation efficiency was calculated by densitometry (A). Data are mean ± sem, n ≥ 3. Data was analysed by ANOVA with a Dunnett's post-hoc test, no significant differences were found.

Having established that the partially-esterified polymers could solubilise lipids effectively, the next step was to examine the solubilisation of membrane proteins. As shown in Fig. 3A, SMA 2000 was able to solubilise 51.5 ± 5.9% of the membrane tether protein ZipA and 55.1 ± 5.4% of the ABC transporter BmrA from *E. coli* membranes, which is comparable to previous studies [22], [23]. Each of the other polymers tested was able to solubilise a comparable amount of these proteins. To show this was also applicable to other membrane protein families and expression systems, we also tested i) extraction of the secondary active transporters LeuT (Fig. 3B) and GltpH (Supplementary Fig. 5) expressed in *E. coli*, ii) the ABC transporter MRP4/ABCC4 expressed in *Sf*9 insect cells (Fig. 3C) and iii) the tetraspanin CD81 expressed in *P. pastoris* (Supplementary Fig. 5). Solubilisation efficiency was higher for MRP4/ABCC4 from *Sf*9 cells, than observed for the microbial expression systems, but there were no obvious differences between the polymers.

### 3.3 Purification of membrane proteins

**Fig. 4:**
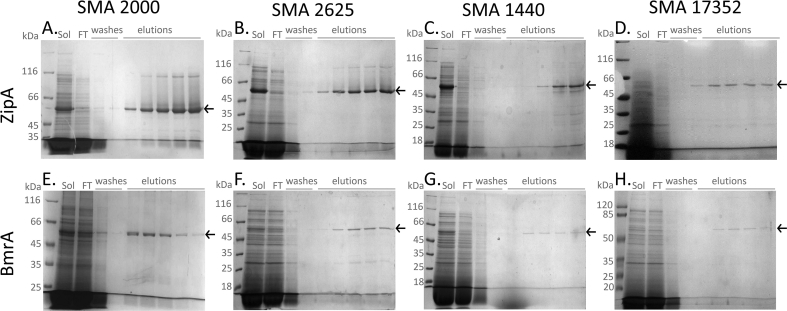
Membrane proteins solubilised with partially esterified polymers can be purified by affinity chromatography. Polymer solubilised membranes (Sol) from *E. coli* expressing ZipA (A–D) or BmrA (E–H) were mixed with HisPur Ni-NTA resin overnight at 4 °C. The sample was transferred to an empty gravity flow column and the flow-through (FT) collected. The resin was washed with 50 bv buffer 1 supplemented with 20 mM imidazole and 20 bv buffer 1 supplemented with 40 mM imidazole. Protein was eluted in 6 fractions of ½ bv with buffer 1 supplemented with 200 mM imidazole. Samples of the solubilised membrane (Sol), flow-through (FT), first and last washes and first 5 elution fractions were run on SDS-PAGE and stained with InstantBlue. These are representative images of ≥4 repeats.

Following effective solubilisation, the next step was to purify the polymer-encapsulated proteins. Ni-NTA affinity purification was carried out as illustrated by the SDS-PAGE images in Fig. 4.

**Fig. 5:**
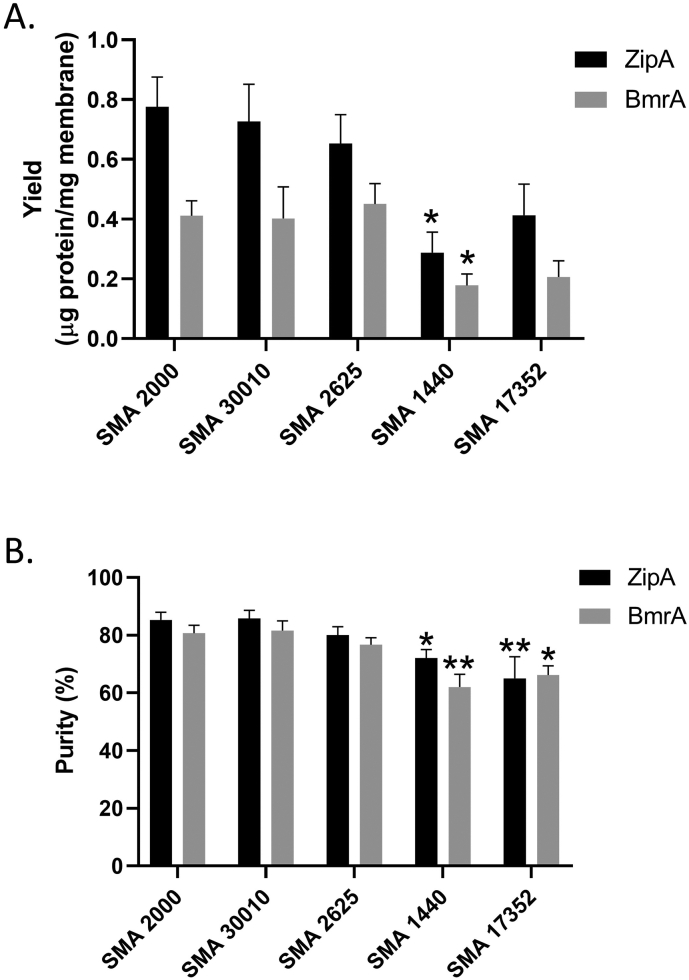
Yield and purity of purified proteins is decreased with some partially esterified polymers. A; Affinity purified protein was quantified by densitometric analysis of samples run on SDS-PAGE alongside BSA standards (0.25–1.5 μg). B; The degree of purity was analysed by densitometric analysis of a single lane of purified protein run on SDS-PAGE. Data are mean ± sem, n ≥ 4. Data were analysed by ANOVA using Dunnett's post-hoc test. *p < 0.05, **p < 0.01 significantly different to SMA 2000.

It can be seen from Fig. 4 that it was possible to purify both the membrane tether protein ZipA and the ABC transporter BmrA by affinity chromatography, with all of the polymers. However, the data in panels C, D, G & H show that the intensity of the protein bands in the elution fractions is lower when using SMA 1440 and possibly also with SMA 17352. This is confirmed by the average results for protein yield shown in Fig. 5A. SMA 2000 gives a yield of purified ZipA of 0.78 ± 0.11 μg protein/mg membrane, and for BmrA 0.41 ± 0.05 μg protein/mg membrane. No significant differences were seen with SMA 30010 or SMA 2625, but SMA 1440 gave a significantly lower yield of 0.29 ± 0.07 μg protein/mg membrane for ZipA and 0.18 ± 0.04 μg protein/mg membrane for BmrA. Furthermore, when analysing the degree of purity, SMA 1440 and SMA 17352 gave a significantly lower degree of purity for both ZipA and BmrA than SMA 2000 (Fig. 5B). Comparable results were also observed for the purification of the secondary active transporters LeuT and GltpH and the tetraspanin CD81 (Supplementary Fig. 5).

### 3.4 Magnesium ion sensitivity

**Fig. 6:**
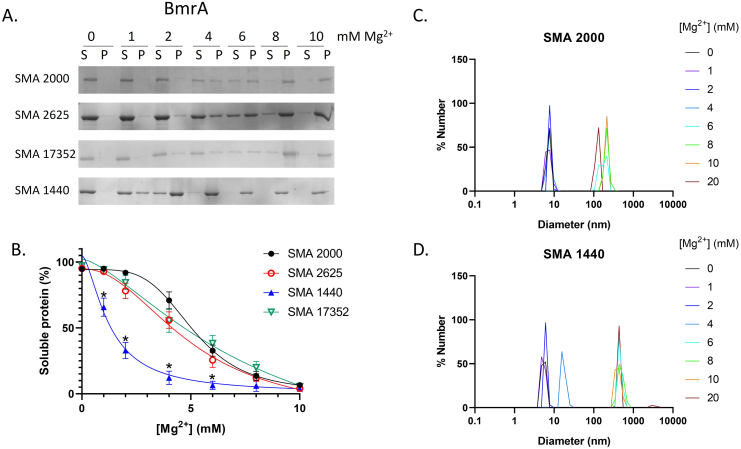
SMA 1440 is more sensitive to magnesium than SMA 2000. A; Representative images of the magnesium ion sensitivity assay on protein-containing SMALPs. BmrA purified with each polymer was mixed with varying concentrations of MgCl_2_ (0–10 mM) and ultracentrifuged (100,000*g*, 20 min, 4 °C). Protein remaining soluble in the supernatant (S) was harvested, and the insoluble material in the pellet (P) resuspended in an equal volume of buffer 1. Samples of both supernatant and pellet were run on SDS-PAGE and stained with InstantBlue. B; SDS-PAGE images such as in A were analysed by densitometry to determine the percentage of protein remaining soluble at each concentration of magnesium. Data are mean ± sem, n ≥ 4, and were fitted with a dose-response curve. Data were analysed by a two-way ANOVA with a Tukey post-hoc test, *p < 0.001 significantly different to SMA 2000. C; Magnesium ion sensitivity assay on lipid-only SMALPs using DLS. Lipid-only SMALPs were prepared and separated from free SMA polymer by size exclusion chromatography. The purified lipid-only SMALPs were mixed with MgCl_2_ (0–20 mM) and analysed by DLS using a DynaPro Plate Reader III and DYNAMICS software with a laser wavelength of 825.4 nm and a detector angle of 150°. Each sample runs in triplicate. Each measurement consisted of 5 scans of 5 s.

The final step was to determine the magnesium sensitivity of SMALPs formed from each of the different polymers. As shown in Fig. 6, both protein-containing SMALPs and lipid-only SMALPs formed with SMA 2000 start to precipitate out of solution at concentrations of Mg^2+^ ≥4 mM, with complete precipitation at 10 mM Mg^2+^. Comparable effects are seen with SMA 2625 and SMA 17352. However, SMALPs formed with SMA 1440 precipitate at significantly lower concentrations of Mg^2+^, with complete precipitation at 6 mM Mg^2+^.

## 4 Discussion

In this study, we investigated the use of three partially esterified SMA polymer variants for the solubilisation of lipids and membrane proteins, using SMA 2000 and SMA 30010 as controls. Solubilisation of DMPC liposomes showed that all of the partially-esterified polymers were able to rapidly solubilise lipids, and that the particles formed were of comparable size to SMA 2000 at just under 10 nm diameter (Fig. 2). This corresponds to previous reports for SMA 2000 [7], [8], [22], but it should be noted that these discs were formed with lipid only, and there is evidence in the literature that the particles formed with SMA polymers can be larger when incorporating large membrane protein complexes [25]. Given that SMA 1440 has previously been shown to extract the photosystem I complex, it is likely this may also be case for this polymer [44], [46]. All of the polymers were equally capable of solubilising various membrane proteins, from different expression systems (Fig. 3). This contrasts somewhat with previous studies using thylakoid membranes from spinach or from cyanobacteria, where SMA 1440 was considerably more efficient at solubilisation, followed by SMA 30010, SMA 17352, SMA 2625 and then SMA 2000 [42], [43], [44]. The reasons for this difference are not clear, but the thylakoid membranes are very densely packed and contain more galactolipid, so differences in polymer-lipid interactions may be responsible. It has previously been shown that the specific lipid and protein composition of membranes plays a large role in the kinetics of solubilisation [47]. Additionally, the thylakoid studies typically used a higher pH and higher temperatures for solubilisation, and this may have an impact.

Following solubilisation, each of the polymers was used for Ni-NTA affinity purification. SMA 2000 and SMA 30010 gave comparable yields and degrees of purity as previously reported [22], [23]. Notably, SMA 1440 gave a lower yield of purified protein than SMA 2000 or SMA 30010, and both SMA 1440 and SMA 17352 gave a lower degree of purity (Fig. 5). Given that the solubilisation efficiency with SMA 1440 was comparable to the other polymers, it raises the question of where did the protein get lost during purification? One possibility is that the SMA 1440 encapsulated proteins did not bind to the Ni-NTA resin with high affinity. It is known that binding to Ni-NTA resin can be impaired for SMALP encapsulated proteins [16], [17]. However, SDS-PAGE gel analysis of the purification does not indicate there is an increase in the amount of target protein in the flow-through (Fig. 4). Another possibility, given the increased sensitivity to divalent cations for SMA 1440, could be that the SMALPs precipitate when interacting with the Ni^2+^ component of the resin, and form aggregates that remain bound to the resin. It would be interesting to test alternative affinity purification approaches with SMA 1440 extracted proteins. The reason for the lower degree of purity obtained with SMA 1440 and SMA 17352 is also not clear. Lower purity has previously been reported when using the polymer DIBMA, and this was suggested to be due to the larger disc size formed by DIBMA which could facilitate co-extraction of more than one protein [40]. However, the DLS data in Fig. 2 show that SMA 1440 and SMA 17352 form discs of comparable size to SMA 2000, at least for lipid only solubilisation. The presence of other proteins could therefore be a non-specific binding to the lipid or polymer.

Finally, we established that SMA 1440 is more sensitive to Mg^2+^ than SMA 2000 (Fig. 6), thus our hypothesis that esterification of some maleic acid groups might improve tolerance was not correct. This agrees with a recent study showing that increasing levels of esterification of SMA with butoxyethanol causes increased sensitivity to Mg^2+^ [46]. In contrast, the modification of a 1:1 styrene:maleic acid polymer by reaction of methylamine with the maleic anhydride, forming an amide derivative (SMAma) rather than an ester, does indeed show increased tolerance to divalent cations [48]. Overall, it seems that the divalent cation sensitivity is more complex than previously thought. DIBMA, for example, has an increased tolerance to divalent cations despite still having the maleic acid groups [35], [40]. In fact the presence of divalent cations has been reported to improve extraction efficiency with DIBMA [49]. There is a suggestion it is linked to the overall hydrophobicity of each polymer. The 3:1 styrene:maleic acid polymers are more hydrophobic than the 2:1 polymers and were shown to be more sensitive to divalent cations [22]. DIBMA has a 1:1 ratio of diisobutylene:maleic acid, so is more hydrophilic overall, and is more tolerant to divalent cations. Similarly, the SMAma has a 1:1 ratio of styrene:maleic acid. However, there is also a fine balance with the hydrophilicity and whether the polymer works for membrane solubilisation, as the 1:1 styrene:maleic acid polymer SMA 1000 was shown to be ineffective [22]. Exactly how and why the divalent cations interact with these polymers to cause the precipitation is not clear. One possibility is simply that they neutralise the overall charge. Although monovalent cations such as Na^+^ are tolerated at very high concentrations (500 mM–1 M), the affinity of carboxyl groups for monovalent cations is much lower than for divalent cations [50]. Alternatively, it may be that differences in coordination chemistry between Na^+^ and Mg^2+^, and something specific to the orientation of the bonds formed between the polymer and Mg^2+^, are responsible for this observation.

In conclusion, partially esterified polymers can solubilise both lipids and membrane proteins effectively, and for more complex, less standard membranes they appear to be superior. However, there are challenges with Ni-NTA affinity chromatography, and SMA 1440 is even more sensitive to divalent cations than the standard SMA 2000.

## Abbreviations

ABC: ATP Binding Cassette
ATR-FTIR: attenuated total reflection – Fourier transform infra-red spectroscopy
BSA: bovine serum albumin
bv: bed volume
CMC: critical micelle concentration
DIBMA: diisobutylene maleic acid
DLS: dynamic light scattering
DMPC: 1,2-dimyristoyl-sn-glycero-3-phosphocholine
GPCR: G protein-coupled receptor
MRP4: multidrug resistance protein 4
Ni-NTA: nickel nitrilotriacetic acid
NMR: nuclear magnetic resonance
Sf9: *Spodoptera frugiperda* cell line
SMA: styrene maleic acid
SMALP: SMA lipid particle
SMAma: SMA methylamine
SMA-QA: styrene maleimide quaternary ammonium
SMI: styrene maleimide

## CRediT authorship contribution statement

**Olivia P. Hawkins**: Investigation, Analysis. **Christine Parisa T. Jahromi**: Investigation, Analysis. **Aiman A. Gulamhussein**: Investigation, Analysis, Writing – original draft. **Stephanie Nestorow**: Investigation, Analysis, Writing – original draft. **Taranpreet Bahra**: Investigation. **Christian Shelton**: Investigation. **Quincy K. Owusu-Mensah**: Investigation. **Naadiya Mohiddin**: Investigation. **Hannah O'Rourke**: Investigation. **Mariam Ajmal**: Investigation. **Kara Byrnes**: Investigation. **Madiha Khan**: Investigation. **Nila N. Nahar**: Investigation. **Arcella Lim**: Investigation. **Cassandra Harris**: Investigation. **Hannah Healy**: Investigation. **Syeda W. Hasan**: Investigation. **Asma Ahmed**: Investigation. **Lora Evans**: Investigation. **Afroditi Vaitsopoulou**: Investigation. **Aneel Akram**: Investigation, Validation. **Chris Williams**: Investigation. **Johanna Binding**: Investigation. **Rumandeep K. Thandi**: Investigation. **Aswathy Joby**: Investigation. **Ashley Guest**: Investigation. **Mohammad Z. Tariq**: Investigation. **Farah Rasool**: Investigation. **Luke Cavanagh**: Investigation. **Simran Kang**: Investigation. **Biser Asparuhov**: Analysis. **Aleksandr Jestin**: Analysis. **Timothy R. Dafforn**: Resources, Supervision, Writing – original draft, Funding acquisition. **John Simms**: Supervision, Writing – original draft. **Roslyn M. Bill**: Supervision, Writing – original draft, Funding acquisition. **Alan D. Goddard**: Supervision, Writing – original draft, Funding acquisition. **Alice J Rothnie**: Conceptualization, Analysis, Validation, Resources, Writing – original draft, Supervision, Project administration, Funding acquisition.

## Declaration of competing interest

The authors declare that they have no known competing financial interests or personal relationships that could have appeared to influence the work reported in this paper.

## Appendix A Supplementary data

Supplementary figures Image 1
