# Supplementary material for: Membrane protein extraction and purification using partially-esterified SMA polymers
Source: Biochim Biophys Acta Biomembr. 2021 Dec 1;1863(12):183758. doi: 10.1016/j.bbamem.2021.183758 (PMC8484863; doi:10.1016/j.bbamem.2021.183758)
Supplement: Supplementary file 1 — Supplementary figures [file mmc1.pdf]

Supplementary Information

Membrane protein extraction and purification using partially-esterified SMA polymers

Olivia P. Hawkins[1*], Christine Parisa T. Jahromi[1*], Aiman A. Gulamhussein[1*], Stephanie Nestorow[2], Taranpreet Bahra[1], Christian Shelton[1], Quincy K. Owusu-Mensah[1], Naadiya Mohiddin[1], Hannah O'Rourke[1], Mariam Ajmal[1], Kara Byrnes[1], Madiha Khan[1], Nila N. Nahar[1], Arcella Lim[1], Cassandra Harris[1], Hannah Healy[1], Syeda W. Hasan[1], Asma Ahmed[1], Lora Evans[1], Afroditi Vaitsopoulou[1], Aneel Akram[1], Chris Williams[1], Johanna Binding[1], Rumandeep K. Thandi[1], Aswathy Joby[1], Ashley Guest[1], Mohammad Z. Tariq[1], Farah Rasool[1], Luke Cavanagh[1], Simran Kang[1], Biser Asparuhov[1], Aleksandr Jestin[1], Timothy R. Dafforn[2], John Simms[1], Roslyn M. Bill[1], Alan D. Goddard[1] & Alice J Rothnie[1**]

[1]College of Health & Life Sciences, Aston University, Aston Triangle, Birmingham, B4 7ET, UK.

[2]School of Biosciences, University of Birmingham, Edgbaston, Birmingham B15 2TT, UK.

Supplementary Figure 1

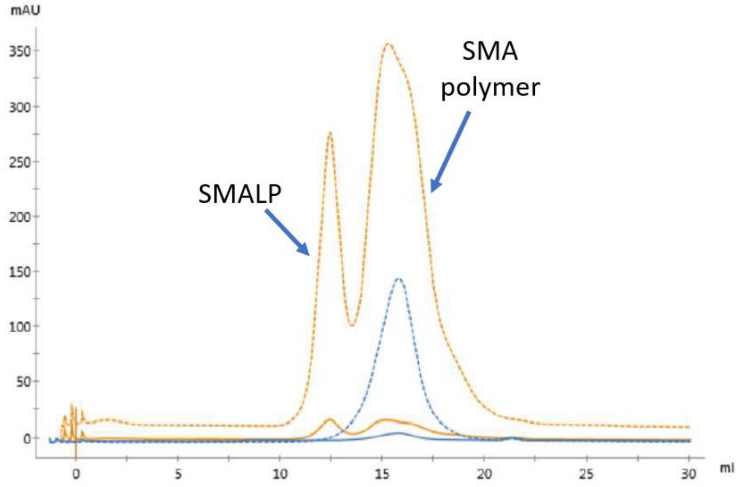

Supplementary Figure 1. Separation of lipid-only SMALPs from free SMA polymer by size exclusion chromatography. *Absorbance traces at 260 nm (dashed lines) or 280 nm (solid lines) from a Superdex 200 10/300 column loaded with either SMA1440 polymer alone (blue) or lipid-only SMALPs formed using SMA1440 (orange).*

Supplementary Figure 2

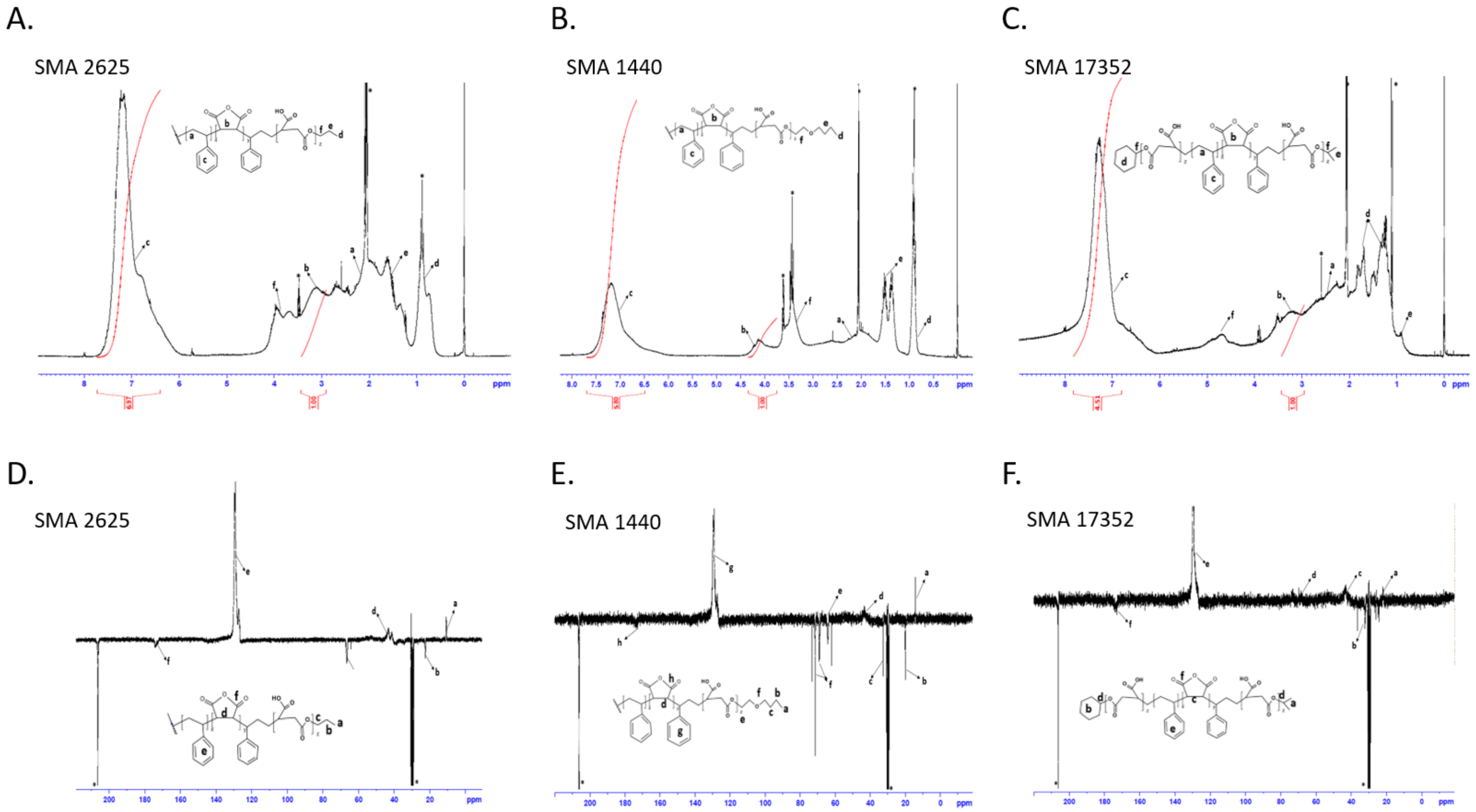

Supplementary Figure 2. NMR spectra for the polymers. *^{1}H-NMR spectra (A-C) and ^{13}C-NMR spectra (D-F) for the partially esterified polymers SMA 2625 (A & D), SMA 1440 (B & E) and SMA 17352 (C & F). The anhydride form of each polymer was dissolved in deuterated acetone and analysed in a 300MHz Brucker NMR spectrometer. Peaks corresponding to the various chemical environments within the structures (inset) have been labelled (a-h).*

Supplementary Figure 3

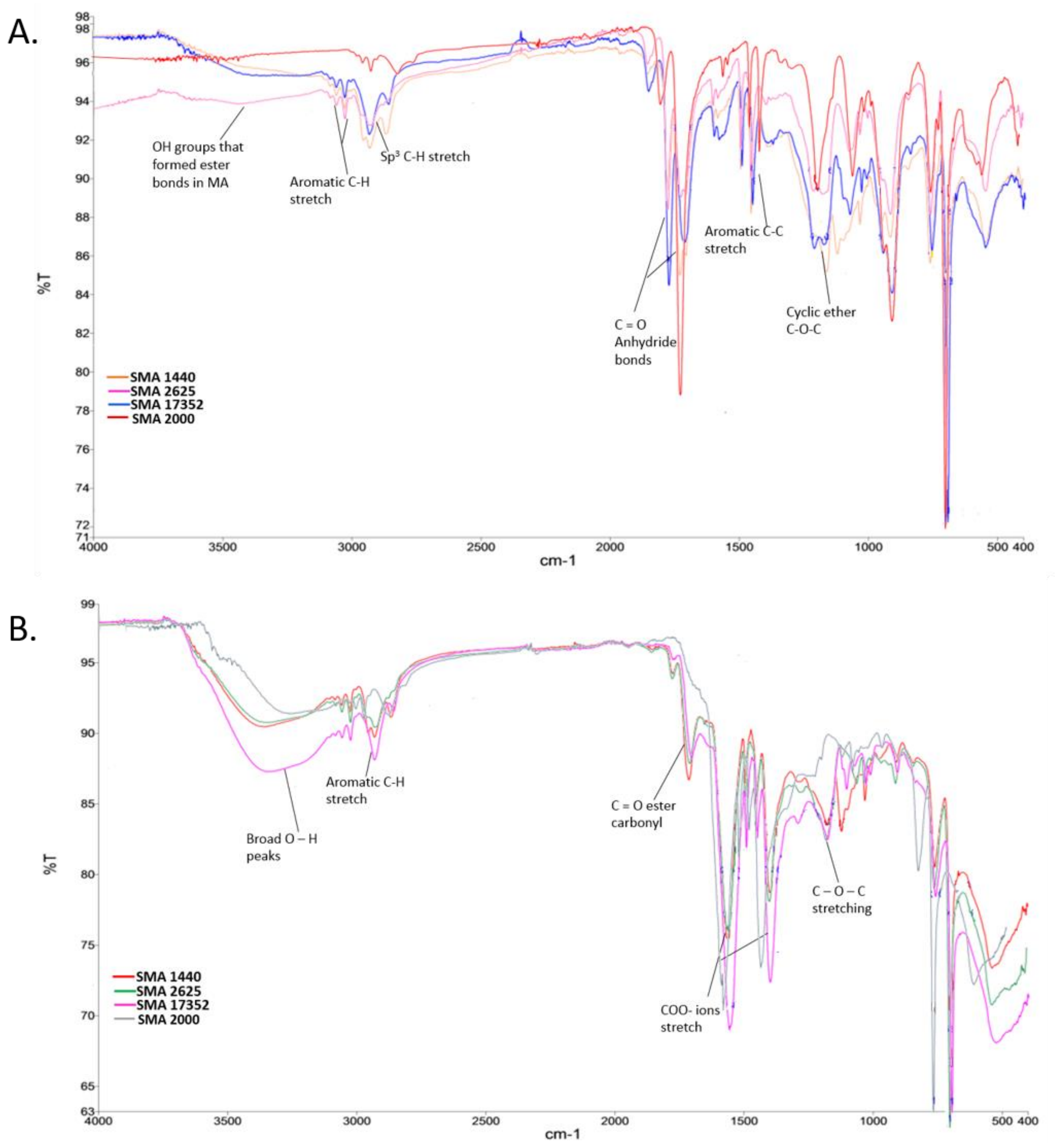

Supplementary Figure 3. FTIR spectra for the polymers. *FTIR spectra for the polymers SMA 2000, SMA 2625, SMA 1440 and SMA 17352, in the pre-hydrolysis anhydride form (A) and post-hydrolysis acid form (B).*

Supplementary Figure 4

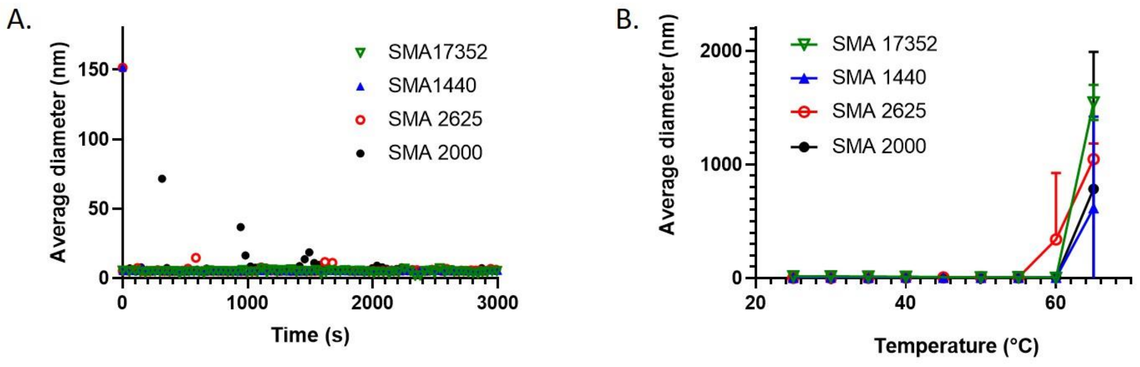

Supplementary Figure 4. Analysis of the stability of SMALP formed from each polymer *Lipid-only SMALPs formed with SMA 2000 (black circle), SMA 2625 (red open circle), SMA 1440 (blue triangle) and SMA 17352 (open green triangle) were analysed by DLS using a DynaPro Plate Reader III and DYNAMICS software with a laser wavelength of 825.4 nm and a detector angle of 150°. A; For time course studies, 100 measurements consisting of 5 acquisitions of 5 s were carried out over the course of 1 hour to monitor lipid-only SMALP stability. Measurements were taken at a temperature of 25 °C. B; For thermostability measurements, purified lipid-only SMALPs were measured. Scans were carried out at a starting point of 25 °C, with discrete 5 °C temperature increments, up to 65 °C. Each increase in temperature was maintained to establish equilibrium before data collection.*

Supplementary Figure 5

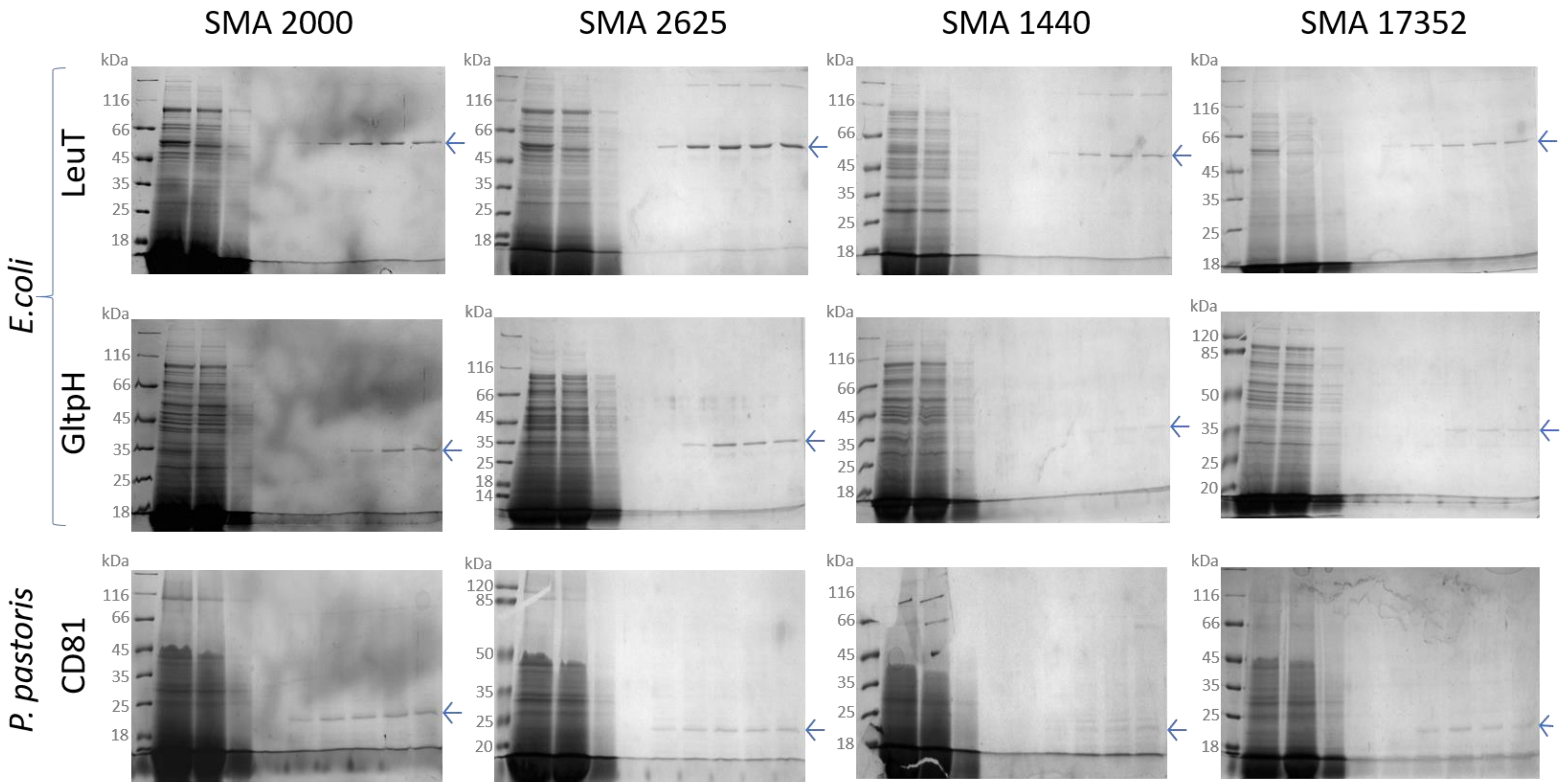

Supplementary Figure 5. Partially esterified SMA polymers can be used for affinity purification of various membrane protein families or expression systems. *The secondary active transporters LeuT and GltpH were expressed in* E. coli*, and the tetraspanin CD81 in* P. pastoris*. Membranes were solubilised with SMA polymers and purified by Ni-NTA affinity chromatography. Samples were analysed by SDS-PAGE and stained with InstantBlue.*
